# 2-Hydroxy­benzoic acid–purin-6-amine (3/1)

**DOI:** 10.1107/S1600536809025240

**Published:** 2009-07-08

**Authors:** Lian-cai Du, Wu-lan Zeng, Xue-ying Liu, Fang-Fang Jian

**Affiliations:** a Microscale Science Institute, Department of Biological Engineering, Weifang University, Weifang 261061, People’s Republic of China; b Microscale Science Institute, Department of Chemistry and Chemical Engineering, Weifang University, Weifang 261061, People’s Republic of China; c Microscale Science Institute, Weifang University, Weifang 261061, People’s Republic of China

## Abstract

In the title 3:1 adduct, 3C_7_H_6_O_3_·C_5_H_5_N_5_, an intra­molecular O—H⋯O hydrogen bond occurs in each of the three 2-hydroxy­benzoic acid mol­ecules. In the crystal, the components are linked by N—H⋯O and O—H⋯N hydrogen bonds.

## Related literature

For medicinal background, see: Forsythe & Ennis (1999 ▶).
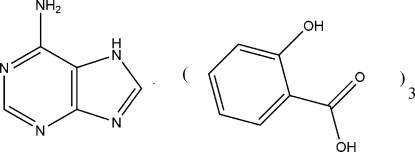


## Experimental

### 

#### Crystal data

3C_7_H_6_O_3_·C_5_H_5_N_5_
*M* *_r_* = 549.49
Monoclinic, 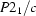

*a* = 10.998 (2) Å
*b* = 10.053 (2) Å
*c* = 23.490 (7) Å
β = 106.98 (3)°
*V* = 2483.9 (10) Å^3^
*Z* = 4
Mo *K*α radiation
μ = 0.11 mm^−1^
*T* = 293 K
0.38 × 0.22 × 0.14 mm

#### Data collection

Siemens SMART CCD diffractometer
Absorption correction: none
22329 measured reflections
5680 independent reflections
4872 reflections with *I* > 2σ(*I*)
*R*_int_ = 0.020

#### Refinement

*R*[*F* ^2^ > 2σ(*F* ^2^)] = 0.041
*wR*(*F* ^2^) = 0.116
*S* = 1.06
5680 reflections
361 parameters
H-atom parameters constrained
Δρ_max_ = 0.83 e Å^−3^
Δρ_min_ = −0.30 e Å^−3^

### 

Data collection: *SMART* (Siemens, 1996 ▶); cell refinement: *SAINT* (Siemens, 1996 ▶); data reduction: *SAINT*; program(s) used to solve structure: *SHELXS97* (Sheldrick, 2008 ▶); program(s) used to refine structure: *SHELXL97* (Sheldrick, 2008 ▶); molecular graphics: *SHELXTL* (Sheldrick, 2008 ▶); software used to prepare material for publication: *SHELXTL*.

## Supplementary Material

Crystal structure: contains datablocks global, I. DOI: 10.1107/S1600536809025240/hb5001sup1.cif

Structure factors: contains datablocks I. DOI: 10.1107/S1600536809025240/hb5001Isup2.hkl

Additional supplementary materials:  crystallographic information; 3D view; checkCIF report

## Figures and Tables

**Table 1:** Hydrogen-bond geometry (Å, °)

*D*—H⋯*A*	*D*—H	H⋯*A*	*D*⋯*A*	*D*—H⋯*A*
O1—H1*D*⋯O3	0.82	1.87	2.5946 (17)	146
O4—H4*B*⋯O5	0.82	1.89	2.6111 (18)	146
O7—H7*A*⋯O9	0.82	1.89	2.6118 (15)	146
O2—H2*C*⋯N4^i^	0.82	1.87	2.6795 (18)	167
O6—H6*B*⋯N3^ii^	0.82	1.82	2.6305 (18)	172
O8—H8*B*⋯N2^iii^	0.82	1.78	2.5864 (17)	168
N1—H1*A*⋯O9^iii^	0.86	2.09	2.9302 (17)	167
N1—H1*B*⋯O3^i^	0.86	2.01	2.8593 (18)	171
N5—H5*A*⋯O7^ii^	0.86	2.14	2.8585 (17)	141

Symmetry codes: (i) 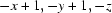; (ii) 
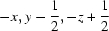
; (iii) 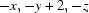.

**Fig. 1.**
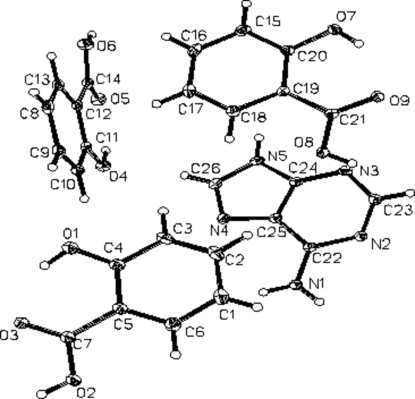
The molecular structure of (I), drawn with 30% probability ellipsoids.

## supplementary crystallographic information

### Comment

Adenine and its derivatives are an important class of compounds because they exhibit better pharmacological activities such as penicillins,antibiotics (Forsythe & Ennis, 1999). We report here the synthesis and structure of the title compound, (I) (Fig. 1), as part of our ongoing studies on new adenine compounds with higher bioactivity.

The adenine ring system is essentially planar, with a dihedral angle of 0.37 (8) between the ring (atoms N4/N5/C24—C26) and the ring (N2/N3/C22—C25). The dihedral angles between the mean planes of the adenine system and rings (C1—C6) and rings (C8—C13) and rings (C15—C20) are 2.41 (7) and 85.83 (7)and 80.3 (7), respectively. The dihedral angle between rings(C1—C6) and rings (C8—C13) is 84.02 (8). In the crystal structure, weak inter molecular C—H···O hydrogen bonds and intramolecular O—H···O hydrogen-bond interactions to stabilize the crystal structure (Table 1). The packing (Fig.2) is further stabilized by weak O—H···O interactions.

### Experimental

Adenine 1.35 g(0.01 mol) and 2-hydroxybenzoic acid 4.14 g(0.03 mol) with ethanol were stirred for 18 h at 353 K. The solution was then filtered and concentrated to afford the white title compound 3.63 g (yield 70%). Colourless blocks of (I) were obtained by slow evaporation of an ethanol-water (10:1 *v*/*v*) solution at room temperature over a period of one week.

### Refinement

The H atoms were located geometrically (C—H = 0.93–0.97 Å, N—H = 0.86Å, O—H = 0.82Å) and refined as riding with *U*_iso_(H) = 1.2 *U*_eq_(carrier). The highest difference peak is 0.45Å from H6A and might indicate unmodelled positional disorder of O1.

### Crystal data

**Table:**

3C_7_H_6_O_3_·C_5_H_5_N_5_	*F*(000) = 1144
*M**_r_* = 549.49	*D*_x_ = 1.469 Mg m^−^^3^
Monoclinic, *P*2_1_/*c*	Mo *K*α radiation, λ = 0.71073 Å
Hall symbol: -P 2ybc	Cell parameters from 5680 reflections
*a* = 10.998 (2) Å	θ = 3.0–27.5°
*b* = 10.053 (2) Å	µ = 0.11 mm^−^^1^
*c* = 23.490 (7) Å	*T* = 293 K
β = 106.98 (3)°	Block, colourless
*V* = 2483.9 (10) Å^3^	0.38 × 0.22 × 0.14 mm
*Z* = 4	

### Data collection

**Table:**

Siemens SMART CCD diffractometer	4872 reflections with *I* > 2σ(*I*)
Radiation source: fine-focus sealed tube	*R*_int_ = 0.020
graphite	θ_max_ = 27.5°, θ_min_ = 3.0°
ω scans	*h* = −14→14
22329 measured reflections	*k* = −13→12
5680 independent reflections	*l* = −30→30

### Refinement

**Table:**

Refinement on *F*^2^	Primary atom site location: structure-invariant direct methods
Least-squares matrix: full	Secondary atom site location: difference Fourier map
*R*[*F*^2^ > 2σ(*F*^2^)] = 0.041	Hydrogen site location: inferred from neighbouring sites
*wR*(*F*^2^) = 0.116	H-atom parameters constrained
*S* = 1.06	*w* = 1/[σ^2^(*F*_o_^2^) + (0.0624*P*)^2^ + 1.1319*P*] where *P* = (*F*_o_^2^ + 2*F*_c_^2^)/3
5680 reflections	(Δ/σ)_max_ < 0.001
361 parameters	Δρ_max_ = 0.83 e Å^−^^3^
0 restraints	Δρ_min_ = −0.30 e Å^−^^3^

### Special details

**Table:**

Geometry. All e.s.d.'s (except the e.s.d. in the dihedral angle between two l.s. planes) are estimated using the full covariance matrix. The cell e.s.d.'s are taken into account individually in the estimation of e.s.d.'s in distances, angles and torsion angles; correlations between e.s.d.'s in cell parameters are only used when they are defined by crystal symmetry. An approximate (isotropic) treatment of cell e.s.d.'s is used for estimating e.s.d.'s involving l.s. planes.
Refinement. Refinement of *F*^2^ against ALL reflections. The weighted *R*-factor *wR* and goodness of fit *S* are based on *F*^2^, conventional *R*-factors *R* are based on *F*, with *F* set to zero for negative *F*^2^. The threshold expression of *F*^2^ > σ(*F*^2^) is used only for calculating *R*-factors(gt) *etc*. and is not relevant to the choice of reflections for refinement. *R*-factors based on *F*^2^ are statistically about twice as large as those based on *F*, and *R*- factors based on ALL data will be even larger.

### Fractional atomic coordinates and isotropic or equivalent isotropic displacement parameters (Å^2^)

**Table:**

	*x*	*y*	*z*	*U*_iso_*/*U*_eq_	
O1	0.58987 (11)	0.54268 (12)	0.10882 (5)	0.0328 (3)	
H1D	0.6464	0.5035	0.0993	0.049*	
O2	0.70858 (10)	0.59642 (10)	−0.04308 (4)	0.0222 (2)	
H2C	0.7663	0.5468	−0.0451	0.033*	
O3	0.73581 (9)	0.48786 (10)	0.04287 (4)	0.0216 (2)	
C1	0.42191 (15)	0.83244 (16)	−0.01407 (8)	0.0309 (3)	
H1C	0.3832	0.8986	−0.0410	0.037*	
C2	0.38415 (14)	0.81020 (16)	0.03701 (7)	0.0290 (3)	
H2B	0.3200	0.8621	0.0440	0.035*	
C3	0.44049 (14)	0.71264 (16)	0.07710 (7)	0.0258 (3)	
H3B	0.4136	0.6985	0.1106	0.031*	
C4	0.53780 (13)	0.63482 (14)	0.06763 (6)	0.0207 (3)	
C5	0.57687 (13)	0.65547 (14)	0.01619 (6)	0.0190 (3)	
C6	0.51750 (14)	0.75499 (15)	−0.02417 (7)	0.0254 (3)	
H6A	0.5427	0.7691	−0.0582	0.030*	
C7	0.67994 (13)	0.57290 (14)	0.00646 (6)	0.0184 (3)	
O4	0.24665 (11)	0.29192 (11)	0.20033 (5)	0.0290 (2)	
H4B	0.2049	0.2650	0.2217	0.043*	
O5	0.18452 (10)	0.26468 (11)	0.29882 (5)	0.0279 (2)	
O6	0.27892 (10)	0.39746 (11)	0.37517 (4)	0.0254 (2)	
H6B	0.2255	0.3646	0.3891	0.038*	
C8	0.54365 (13)	0.53292 (16)	0.29552 (7)	0.0242 (3)	
H8A	0.6111	0.5862	0.3164	0.029*	
C9	0.52558 (14)	0.50464 (17)	0.23539 (7)	0.0279 (3)	
H9A	0.5809	0.5405	0.2162	0.033*	
C10	0.42685 (15)	0.42432 (17)	0.20407 (7)	0.0277 (3)	
H10A	0.4161	0.4064	0.1641	0.033*	
C11	0.34292 (13)	0.36986 (15)	0.23260 (6)	0.0222 (3)	
C12	0.35880 (13)	0.39957 (14)	0.29283 (6)	0.0189 (3)	
C13	0.46011 (13)	0.48074 (14)	0.32360 (6)	0.0202 (3)	
H13A	0.4713	0.4998	0.3635	0.024*	
C14	0.26650 (13)	0.34705 (14)	0.32213 (6)	0.0199 (3)	
N1	0.08652 (11)	0.72297 (12)	−0.04856 (5)	0.0205 (2)	
H1A	0.0719	0.7725	−0.0797	0.025*	
H1B	0.1424	0.6607	−0.0429	0.025*	
N2	−0.06434 (11)	0.84210 (12)	−0.01967 (5)	0.0193 (2)	
N3	−0.12090 (11)	0.80213 (12)	0.06983 (5)	0.0208 (2)	
N4	0.11815 (11)	0.56210 (12)	0.06827 (5)	0.0194 (2)	
N5	0.00044 (11)	0.61900 (12)	0.12747 (5)	0.0200 (2)	
H5A	−0.0296	0.6176	0.1574	0.024*	
C22	0.02299 (12)	0.74279 (14)	−0.00930 (6)	0.0173 (3)	
C23	−0.12982 (13)	0.86541 (15)	0.01934 (6)	0.0218 (3)	
H23A	−0.1886	0.9344	0.0098	0.026*	
C24	−0.03382 (12)	0.70387 (14)	0.08015 (6)	0.0176 (3)	
C25	0.03992 (12)	0.66798 (14)	0.04351 (5)	0.0169 (3)	
C26	0.09105 (13)	0.53725 (15)	0.11826 (6)	0.0210 (3)	
H26A	0.1301	0.4703	0.1446	0.025*	
O7	−0.00080 (10)	1.02622 (10)	0.25745 (4)	0.0227 (2)	
H7A	−0.0214	1.0709	0.2269	0.034*	
O8	0.13258 (9)	0.99434 (10)	0.10831 (4)	0.0209 (2)	
H8B	0.1022	1.0483	0.0818	0.031*	
O9	0.00284 (10)	1.09961 (10)	0.15130 (4)	0.0226 (2)	
C15	0.13464 (15)	0.84945 (15)	0.30203 (6)	0.0243 (3)	
H15A	0.1073	0.8584	0.3357	0.029*	
C16	0.22228 (14)	0.75286 (15)	0.30017 (7)	0.0258 (3)	
H16A	0.2539	0.6973	0.3328	0.031*	
C17	0.26428 (14)	0.73742 (15)	0.24987 (7)	0.0238 (3)	
H17A	0.3225	0.6712	0.2486	0.029*	
C18	0.21830 (13)	0.82174 (14)	0.20201 (6)	0.0193 (3)	
H18A	0.2467	0.8124	0.1686	0.023*	
C19	0.12974 (12)	0.92108 (13)	0.20291 (6)	0.0161 (3)	
C20	0.08674 (13)	0.93391 (14)	0.25345 (6)	0.0182 (3)	
C21	0.08283 (12)	1.01232 (13)	0.15196 (6)	0.0170 (3)	

### Atomic displacement parameters (Å^2^)

**Table:**

	*U*^11^	*U*^22^	*U*^33^	*U*^12^	*U*^13^	*U*^23^
O1	0.0346 (6)	0.0406 (7)	0.0266 (5)	0.0103 (5)	0.0144 (5)	0.0040 (5)
O2	0.0233 (5)	0.0252 (5)	0.0210 (5)	0.0045 (4)	0.0110 (4)	0.0023 (4)
O3	0.0221 (5)	0.0234 (5)	0.0204 (5)	0.0038 (4)	0.0081 (4)	0.0015 (4)
C1	0.0284 (8)	0.0248 (8)	0.0379 (9)	0.0063 (6)	0.0070 (7)	0.0024 (6)
C2	0.0219 (7)	0.0259 (8)	0.0389 (9)	0.0027 (6)	0.0085 (6)	−0.0100 (6)
C3	0.0223 (7)	0.0301 (8)	0.0268 (7)	−0.0025 (6)	0.0103 (6)	−0.0099 (6)
C4	0.0198 (6)	0.0216 (7)	0.0199 (6)	−0.0024 (5)	0.0047 (5)	−0.0045 (5)
C5	0.0177 (6)	0.0183 (6)	0.0210 (6)	−0.0020 (5)	0.0058 (5)	−0.0039 (5)
C6	0.0250 (7)	0.0234 (7)	0.0279 (7)	0.0016 (6)	0.0081 (6)	0.0018 (6)
C7	0.0173 (6)	0.0192 (6)	0.0188 (6)	−0.0034 (5)	0.0052 (5)	−0.0031 (5)
O4	0.0315 (6)	0.0307 (6)	0.0236 (5)	0.0018 (5)	0.0063 (4)	−0.0038 (4)
O5	0.0272 (5)	0.0261 (6)	0.0307 (6)	−0.0055 (5)	0.0092 (4)	0.0005 (4)
O6	0.0258 (5)	0.0320 (6)	0.0235 (5)	−0.0064 (4)	0.0149 (4)	0.0004 (4)
C8	0.0177 (6)	0.0276 (8)	0.0283 (7)	0.0040 (6)	0.0083 (5)	0.0080 (6)
C9	0.0233 (7)	0.0367 (9)	0.0288 (7)	0.0096 (6)	0.0156 (6)	0.0135 (6)
C10	0.0307 (8)	0.0361 (9)	0.0196 (7)	0.0123 (7)	0.0123 (6)	0.0061 (6)
C11	0.0229 (7)	0.0225 (7)	0.0209 (6)	0.0081 (6)	0.0059 (5)	0.0021 (5)
C12	0.0202 (6)	0.0193 (7)	0.0187 (6)	0.0060 (5)	0.0082 (5)	0.0048 (5)
C13	0.0200 (6)	0.0219 (7)	0.0194 (6)	0.0046 (5)	0.0069 (5)	0.0051 (5)
C14	0.0196 (6)	0.0189 (6)	0.0219 (6)	0.0031 (5)	0.0072 (5)	0.0047 (5)
N1	0.0242 (6)	0.0239 (6)	0.0162 (5)	0.0061 (5)	0.0101 (4)	0.0050 (4)
N2	0.0218 (5)	0.0202 (6)	0.0172 (5)	0.0020 (5)	0.0074 (4)	0.0020 (4)
N3	0.0220 (6)	0.0245 (6)	0.0181 (5)	0.0007 (5)	0.0096 (4)	−0.0012 (4)
N4	0.0188 (5)	0.0209 (6)	0.0183 (5)	0.0004 (5)	0.0052 (4)	0.0019 (4)
N5	0.0215 (6)	0.0271 (6)	0.0128 (5)	−0.0049 (5)	0.0075 (4)	−0.0006 (4)
C22	0.0174 (6)	0.0189 (6)	0.0157 (6)	−0.0017 (5)	0.0048 (5)	−0.0007 (5)
C23	0.0229 (7)	0.0232 (7)	0.0205 (6)	0.0025 (6)	0.0081 (5)	0.0008 (5)
C24	0.0173 (6)	0.0208 (7)	0.0149 (6)	−0.0048 (5)	0.0051 (5)	−0.0019 (5)
C25	0.0177 (6)	0.0198 (6)	0.0138 (6)	−0.0024 (5)	0.0054 (5)	−0.0006 (5)
C26	0.0201 (6)	0.0238 (7)	0.0174 (6)	−0.0022 (6)	0.0029 (5)	0.0031 (5)
O7	0.0308 (5)	0.0222 (5)	0.0178 (5)	0.0063 (4)	0.0114 (4)	0.0022 (4)
O8	0.0247 (5)	0.0238 (5)	0.0165 (4)	0.0076 (4)	0.0094 (4)	0.0052 (4)
O9	0.0278 (5)	0.0235 (5)	0.0190 (5)	0.0097 (4)	0.0107 (4)	0.0047 (4)
C15	0.0324 (7)	0.0245 (7)	0.0169 (6)	−0.0010 (6)	0.0086 (6)	0.0034 (5)
C16	0.0290 (7)	0.0239 (7)	0.0215 (7)	−0.0003 (6)	0.0025 (6)	0.0089 (5)
C17	0.0208 (6)	0.0202 (7)	0.0288 (7)	0.0025 (6)	0.0048 (5)	0.0045 (6)
C18	0.0181 (6)	0.0192 (7)	0.0211 (6)	−0.0005 (5)	0.0065 (5)	0.0009 (5)
C19	0.0164 (6)	0.0162 (6)	0.0152 (6)	−0.0015 (5)	0.0037 (5)	0.0006 (5)
C20	0.0204 (6)	0.0169 (6)	0.0176 (6)	−0.0019 (5)	0.0059 (5)	−0.0010 (5)
C21	0.0180 (6)	0.0177 (6)	0.0157 (6)	−0.0009 (5)	0.0055 (5)	−0.0005 (5)

### Geometric parameters (Å, °)

**Table:**

O1—C4	1.3411 (18)	N1—C22	1.3247 (17)
O1—H1D	0.8201	N1—H1A	0.8601
O2—C7	1.3128 (17)	N1—H1B	0.8599
O2—H2C	0.8200	N2—C23	1.3411 (18)
O3—C7	1.2381 (17)	N2—C22	1.3572 (18)
C1—C6	1.383 (2)	N3—C23	1.3241 (18)
C1—C2	1.398 (2)	N3—C24	1.3476 (19)
C1—H1C	0.9300	N4—C26	1.3169 (18)
C2—C3	1.375 (2)	N4—C25	1.3854 (18)
C2—H2B	0.9300	N5—C26	1.3571 (19)
C3—C4	1.395 (2)	N5—C24	1.3642 (18)
C3—H3B	0.9300	N5—H5A	0.8601
C4—C5	1.4110 (19)	C22—C25	1.4155 (18)
C5—C6	1.402 (2)	C23—H23A	0.9300
C5—C7	1.4756 (19)	C24—C25	1.3916 (18)
C6—H6A	0.9300	C26—H26A	0.9300
O4—C11	1.3568 (19)	O7—C20	1.3600 (17)
O4—H4B	0.8199	O7—H7A	0.8201
O5—C14	1.2289 (18)	O8—C21	1.3082 (16)
O6—C14	1.3146 (17)	O8—H8B	0.8200
O6—H6B	0.8203	O9—C21	1.2394 (17)
C8—C13	1.381 (2)	C15—C16	1.378 (2)
C8—C9	1.397 (2)	C15—C20	1.3964 (19)
C8—H8A	0.9300	C15—H15A	0.9300
C9—C10	1.380 (2)	C16—C17	1.397 (2)
C9—H9A	0.9300	C16—H16A	0.9300
C10—C11	1.401 (2)	C17—C18	1.381 (2)
C10—H10A	0.9300	C17—H17A	0.9300
C11—C12	1.4059 (19)	C18—C19	1.3995 (19)
C12—C13	1.400 (2)	C18—H18A	0.9300
C12—C14	1.4803 (19)	C19—C20	1.4062 (18)
C13—H13A	0.9300	C19—C21	1.4762 (18)
			
C4—O1—H1D	109.5	C22—N1—H1B	120.1
C7—O2—H2C	109.5	H1A—N1—H1B	120.0
C6—C1—C2	119.24 (15)	C23—N2—C22	119.92 (12)
C6—C1—H1C	120.4	C23—N3—C24	112.09 (12)
C2—C1—H1C	120.4	C26—N4—C25	104.22 (11)
C3—C2—C1	120.96 (14)	C26—N5—C24	106.84 (11)
C3—C2—H2B	119.5	C26—N5—H5A	126.6
C1—C2—H2B	119.5	C24—N5—H5A	126.6
C2—C3—C4	120.26 (14)	N1—C22—N2	118.36 (12)
C2—C3—H3B	119.9	N1—C22—C25	124.68 (13)
C4—C3—H3B	119.9	N2—C22—C25	116.96 (12)
O1—C4—C3	117.27 (13)	N3—C23—N2	127.88 (13)
O1—C4—C5	123.15 (13)	N3—C23—H23A	116.1
C3—C4—C5	119.58 (14)	N2—C23—H23A	116.1
C6—C5—C4	119.08 (13)	N3—C24—N5	128.14 (12)
C6—C5—C7	121.60 (13)	N3—C24—C25	126.21 (12)
C4—C5—C7	119.32 (13)	N5—C24—C25	105.65 (12)
C1—C6—C5	120.86 (15)	N4—C25—C24	110.01 (11)
C1—C6—H6A	119.6	N4—C25—C22	133.06 (12)
C5—C6—H6A	119.6	C24—C25—C22	116.93 (12)
O3—C7—O2	122.51 (13)	N4—C26—N5	113.29 (12)
O3—C7—C5	122.02 (12)	N4—C26—H26A	123.4
O2—C7—C5	115.47 (12)	N5—C26—H26A	123.4
C11—O4—H4B	109.5	C20—O7—H7A	109.5
C14—O6—H6B	109.5	C21—O8—H8B	109.5
C13—C8—C9	119.17 (15)	C16—C15—C20	120.21 (13)
C13—C8—H8A	120.4	C16—C15—H15A	119.9
C9—C8—H8A	120.4	C20—C15—H15A	119.9
C10—C9—C8	121.02 (14)	C15—C16—C17	120.81 (13)
C10—C9—H9A	119.5	C15—C16—H16A	119.6
C8—C9—H9A	119.5	C17—C16—H16A	119.6
C9—C10—C11	119.96 (14)	C18—C17—C16	119.17 (14)
C9—C10—H10A	120.0	C18—C17—H17A	120.4
C11—C10—H10A	120.0	C16—C17—H17A	120.4
O4—C11—C10	118.31 (13)	C17—C18—C19	121.12 (13)
O4—C11—C12	122.16 (13)	C17—C18—H18A	119.4
C10—C11—C12	119.52 (14)	C19—C18—H18A	119.4
C13—C12—C11	119.32 (13)	C18—C19—C20	119.04 (12)
C13—C12—C14	121.21 (12)	C18—C19—C21	120.74 (12)
C11—C12—C14	119.45 (13)	C20—C19—C21	120.22 (12)
C8—C13—C12	120.99 (13)	O7—C20—C15	117.65 (12)
C8—C13—H13A	119.5	O7—C20—C19	122.71 (12)
C12—C13—H13A	119.5	C15—C20—C19	119.64 (13)
O5—C14—O6	122.80 (13)	O9—C21—O8	123.07 (12)
O5—C14—C12	123.13 (13)	O9—C21—C19	121.93 (12)
O6—C14—C12	114.06 (12)	O8—C21—C19	114.99 (12)
C22—N1—H1A	119.9		
			
C6—C1—C2—C3	−0.1 (2)	C22—N2—C23—N3	0.3 (2)
C1—C2—C3—C4	0.7 (2)	C23—N3—C24—N5	179.88 (13)
C2—C3—C4—O1	179.11 (14)	C23—N3—C24—C25	−0.5 (2)
C2—C3—C4—C5	−0.9 (2)	C26—N5—C24—N3	179.76 (13)
O1—C4—C5—C6	−179.48 (13)	C26—N5—C24—C25	0.09 (14)
C3—C4—C5—C6	0.5 (2)	C26—N4—C25—C24	−0.25 (15)
O1—C4—C5—C7	−0.1 (2)	C26—N4—C25—C22	179.27 (15)
C3—C4—C5—C7	179.94 (12)	N3—C24—C25—N4	−179.58 (12)
C2—C1—C6—C5	−0.3 (2)	N5—C24—C25—N4	0.10 (15)
C4—C5—C6—C1	0.1 (2)	N3—C24—C25—C22	0.8 (2)
C7—C5—C6—C1	−179.32 (14)	N5—C24—C25—C22	−179.51 (11)
C6—C5—C7—O3	177.52 (13)	N1—C22—C25—N4	−0.4 (2)
C4—C5—C7—O3	−1.9 (2)	N2—C22—C25—N4	180.00 (13)
C6—C5—C7—O2	−2.09 (19)	N1—C22—C25—C24	179.08 (13)
C4—C5—C7—O2	178.54 (12)	N2—C22—C25—C24	−0.50 (18)
C13—C8—C9—C10	−0.8 (2)	C25—N4—C26—N5	0.31 (15)
C8—C9—C10—C11	0.0 (2)	C24—N5—C26—N4	−0.26 (16)
C9—C10—C11—O4	179.69 (13)	C20—C15—C16—C17	−0.2 (2)
C9—C10—C11—C12	1.1 (2)	C15—C16—C17—C18	1.0 (2)
O4—C11—C12—C13	−179.95 (13)	C16—C17—C18—C19	−0.6 (2)
C10—C11—C12—C13	−1.4 (2)	C17—C18—C19—C20	−0.4 (2)
O4—C11—C12—C14	−1.3 (2)	C17—C18—C19—C21	178.90 (13)
C10—C11—C12—C14	177.17 (13)	C16—C15—C20—O7	179.13 (13)
C9—C8—C13—C12	0.5 (2)	C16—C15—C20—C19	−0.9 (2)
C11—C12—C13—C8	0.6 (2)	C18—C19—C20—O7	−178.81 (12)
C14—C12—C13—C8	−177.94 (13)	C21—C19—C20—O7	1.8 (2)
C13—C12—C14—O5	−172.14 (13)	C18—C19—C20—C15	1.2 (2)
C11—C12—C14—O5	9.3 (2)	C21—C19—C20—C15	−178.15 (13)
C13—C12—C14—O6	8.60 (19)	C18—C19—C21—O9	178.77 (13)
C11—C12—C14—O6	−169.98 (12)	C20—C19—C21—O9	−1.9 (2)
C23—N2—C22—N1	−179.60 (13)	C18—C19—C21—O8	−1.68 (18)
C23—N2—C22—C25	0.01 (19)	C20—C19—C21—O8	177.67 (12)
C24—N3—C23—N2	−0.1 (2)		

### Hydrogen-bond geometry (Å, °)

**Table:**

*D*—H···*A*	*D*—H	H···*A*	*D*···*A*	*D*—H···*A*
O1—H1D···O3	0.82	1.87	2.5946 (17)	146
O4—H4B···O5	0.82	1.89	2.6111 (18)	146
O7—H7A···O9	0.82	1.89	2.6118 (15)	146
O2—H2C···N4^i^	0.82	1.87	2.6795 (18)	167
O6—H6B···N3^ii^	0.82	1.82	2.6305 (18)	172
O8—H8B···N2^iii^	0.82	1.78	2.5864 (17)	168
N1—H1A···O9^iii^	0.86	2.09	2.9302 (17)	167
N1—H1B···O3^i^	0.86	2.01	2.8593 (18)	171
N5—H5A···O7^ii^	0.86	2.14	2.8585 (17)	141

Symmetry codes: (i) −*x*+1, −*y*+1, −*z*; (ii) −*x*, *y*−1/2, −*z*+1/2; (iii) −*x*, −*y*+2, −*z*.

## References

[bb1] Forsythe, P. & Ennis, M. (1999). *Inflam. Res.* **48**, 301–307. 10.1007/s000110050464 10442481

[bb2] Sheldrick, G. M. (2008). *Acta Cryst.* A**64**, 112–122. 10.1107/S0108767307043930 18156677

[bb3] Siemens (1996). *SMART* and *SAINT* Siemens Analytical X-ray Instruments Inc., Madison, Wisconsin, USA.

